# Adjuvant TACE inhibitor treatment improves the outcome of TLR2^-/- ^mice with experimental pneumococcal meningitis

**DOI:** 10.1186/1471-2334-7-25

**Published:** 2007-04-11

**Authors:** Hakim Echchannaoui, Stephen L Leib, Ulf Neumann, Regine MA Landmann

**Affiliations:** 1 Division of Infectious Diseases, Department of Research, University Hospitals, Basel, Switzerland; 2 Institute for Infectious Diseases, University of Bern, Switzerland; 3 Novartis Institute for BioMedical Research, Basel, Switzerland

## Abstract

**Background:**

*Streptococcus (S.) pneumoniae *meningitis has a high lethality despite antibiotic treatment. Inflammation is a major pathogenetic factor, which is unresponsive to antibiotics. Therefore adjunctive therapies with antiinflammatory compounds have been developed. TNF484 is a TNF-alpha converting enzyme (TACE) inhibitor and has been found efficacious in experimental meningitis. Toll-like receptor 2 (TLR2) contributes to host response in pneumococcal meningitis by enhancing bacterial clearing and downmodulating inflammation. In this study, TNF484 was applied in mice, which lacked TLR2 and exhibited a strong meningeal inflammation.

**Methods:**

10^3 ^CFU *S. pneumoniae *serotype 3 was inoculated subarachnoidally into C57BL/6 wild type (wt) mice or TLR2^-/-^, CD14^-/- ^and CD14^-/-^/TLR2^-/- ^mice. Severity of disease and survival was followed over 9 days. Response to antibiotics (80 mg/kg ceftriaxone i.p. for 5 days) and/or TACE inhibitor treatment (1 mg/kg s.c. twice daily for 4 days) was evaluated. Animals were sacrificed after 12, 24, and 48 h for analysis of bacterial load in cerebrospinal fluid (CSF) and brain and for TNF and leukocyte measurements in CSF.

**Results:**

TLR2^-/- ^mice were significantly sicker than the other mouse strains 24 h after infection. All knockout mice showed higher disease severity after 48 h and died earlier than wt mice. TNF release into CSF was significantly more elevated in TLR2^-/- ^than in the other strains after 24 h. Brain bacterial numbers were significantly higher in all knockout than wt mice after 24 h. Modulation of outcome by antibiotic and TACE inhibitor treatment was evaluated. With antibiotic therapy all wt, CD14^-/- ^and TLR2^-/-^/CD14^-/- ^mice, but only 79% of TLR2^-/- ^mice, were rescued. TACE inhibitor treatment alone did not rescue, but prolonged survival in wt mice, and in TLR2^-/- ^and CD14^-/- ^mice to the values observed in untreated wt mice. By combined antibiotic and TACE inhibitor treatment 95% of TLR2^-/- ^mice were rescued.

**Conclusion:**

During pneumococcal meningitis strong inflammation in TLR2-deficiency was associated with incomplete responsiveness to antibiotics and complete response to combined antibiotic and TACE inhibitor treatment. TACE inhibitor treatment offers a promising adjuvant therapeutic strategy in pneumococcal meningitis.

## Background

*Streptococcus pneumoniae *is the cause of the most severe and most frequent form of adult bacterial meningitis [1]. Therapy with antibiotics is only partially effective in preventing mortality and development of neurologic sequelae [2]. Inflammation contributes to morbidity and mortality, but is itself unresponsive to antibiotics [3]. New adjunctive therapies reducing inflammatory molecules are needed [4]. In this respect, TNF and matrix-metalloproteases (MMPs) are major therapeutic targets since both molecules are up-regulated early [5]; TNF levels in CSF are related to disease severity [6] and TNF and MMP's contribute to brain injury during pneumococcal meningitis [7-9]. TNF and its receptors are shed from the membrane by TNF alpha converting enzyme (TACE) [7]. TNF484 is a novel water-soluble inhibitor of MMPs and TACE release, which is active *in vitro *[10] and has been found efficacious in experimental rat meningitis [9]. The pattern recognition receptor TLR2 is expressed on resting mouse phagocytes [11] and mediates inflammatory responses to cell wall lipoteichoic acid and membrane lipoproteins of Gram-positive bacteria [12,13]. Live Gram-positive bacteria, while expressing TLR2 ligands on their surface, do not depend on TLR2 for phagocytosis and killing, nor for induction of inflammation; however, TLR2 modulates infection. We and others found accelerated mortality of TLR2^-/-^mice with pneumococcal meningitis [6,14]. Early death was associated with high brain bacterial load, and strong TNF expression in brain infiltrating cells. Excess TNF in TLR2^-/- ^mice was not due to higher infiltrating leukocyte numbers in this strain, but to excess expression of TNF per cell [11,15]. Our resullts indicate that TLR2 functions both to upregulate inflammation in response to bacterial components and to downregulate inflammation elicited by infection with live S. *pneumoniae*. Besides TLR2, CD14 was also recognized as receptor for Gram-positive cell wall components [16,17]. It is poorly expressed in resting mouse phagocytes [18] and strongly induced upon stimulation with bacterial components or infection *in vitro *and *in vivo *[18,19]. This suggests a role in host immune response [20]. The association of CD14 with TLR2 in a heteromeric complex was shown to be required for cell activation by TLR2 ligands [13,21-23], and recently lipopeptide binding to CD14 was found to induce proximity between CD14, TLR2 and TLR1 [21]. In pneumococcal meningitis, we found a shorter survival in CD14^-/- ^than in wt mice, which was due to enhanced CXCR2 expression leading to early recruitment of leukocyte and increased TNF in cerebrospinal fluid (CSF) [18]. From our *in vivo *studies using single knockout mice, it appears that TLR2 and CD14 both are protective in meningitis, although by different mechanisms. Because both strains showed excessive TNF, we compared the treatment response in wt, TLR2^-/-^, CD14^-/-^, and CD14^-/-^/TLR2^-/-^double knockout mice with meningitis to antibiotic treatment and/or anti-inflammatory treatment with TACE inhibitor.

## Methods

### Preparation of bacterial inocula

*Streptococcus pneumoniae *(clinical isolate of serotype 3, H14) were grown for 7 h in double Mueller Hinton broth (MHB) (DIFCO Laboratories Detroit, USA), then subcultured overnight in new MHB, and washed in 0.9% sterile saline (12,000 × g for 6 min) immediately before use. The inoculum size was calculated from optical density (OD) determinations (OD 0.4 = 1 × 10^8 ^CFU/ml) and was retrospectively assessed by CFU counting on blood agar plates.

### Mouse meningitis model

Six to eight week old C57BL/6 (wild type, wt) were bred at the Animal House of the University Hospital Basel. TLR2^-/- ^mice were kindly provided by William J. Rieflin (Tularik, South San Francisco, CA) and CD14^-/- ^mice by Mason Freeman (Massachusetts General Hospital, Boston). Both mouse strains had been backcrossed for 10 generations on a C57BL/6 background. The two strains with targeted gene deletions were crossed to obtain TLR2^-/-^/CD14^-/-^double knockout mice. All animals were kept under specific pathogen free conditions in the Animal House of the Department of Research, University Hospital Basel according to the regulations of the Swiss veterinary law. Mice were anaesthetized via intraperitoneal (i.p.) injection of 100 mg/kg Ketamine (Ketalar^©^; Warner-Lambert AG, Baar, Switzerland) and 20 mg/kg Xylazinum (Xylapan^©^; Graeub AG, Bern, Switzerland), and subsequently subarachnoidally inoculated into the left forebrain with either 0.9% NaCl or live *S. pneumoniae *(2 × 10^2 ^or 3 × 10^3 ^CFU) in a 25 μl volume. The health status of the mice was assessed by the following scores as described previously [7]: (1) normal motor activity and turned upright in <5 s when put on their back; (2) decreased spontaneous activity, but still turned up in <5 s; (3) turned up in >5 s; (4) did not turn up; (5) did not move. After 6, 12, 24, 48 and 72 h or if they presented score 5, mice were sacrificed by i.p. injection of 100 mg/kg pentobarbital (Abbott Laboratories, North Chicago, IL). Animals were perfused with Ringer's solution (Braun Medical AG, Emmenbrücke, Switzerland) into the left cardiac ventricle. Cerebrospinal fluid (CSF) was harvested by puncture of the cisterna magna as described previously [20]. Due to the small volumes (3–6 μl) obtained from each animal, CSF from 3 mice were pooled.

### Determination of bacterial counts and inflammatory parameters

Leukocytes in CSF were counted in a hematocytometer and differentiated by Wright stain on cytospin preparations. Then CSF was serially diluted in 0.9% saline to assess the bacterial load after plating and incubation at 37°C for 24 h. Cell free CSF was obtained by centrifugation at 800 × g for 7 min (room temperature) and samples were stored at -20°C until cytokine determination. For histology, brains were fixed in 4% paraformaldehyde, paraffin embedded and stained with haematoxylin-eosin (H&E). For bacterial counting, brains were homogenized with a Polytron homogenizer in 1 ml of phosphate buffer saline (PBS). Bacterial titers were determined by plating serial 10-fold dilutions in 0.9% saline on blood agar plates. The concentration of the pro-inflammatory cytokine TNF in CSF was determined with a bioassay, measuring the degree of cytotoxicity on WEHI cells in the presence of 1 μg/ml actinomycin D, and with use of mouse rTNF as a standard. Matrix metalloproteinase 9 was determined with zymography from gelatine-sepharose purified, frozen brain homogenates as described previously [8].

### Treatment

Treatments with antibiotic and TACE inhibitor were performed as previously described [6,7]. Briefly, mice were treated with either 80 mg/kg ceftriaxone (Rocephin, Hoffmann-La Roche, Switzerland, dissolved in 0.1 ml saline) via i.p. injection twice daily for 5 days and/or with subcutaneous (s.c.) injection of 1 mg/kg water soluble TACE inhibitor TNF484 twice daily for 4 days. Treatments with TNF484 and antibiotics were started 12 h and 18 h after infection, respectively.

### Statistical analysis

Differences in survival between different mouse strains and treatments were analyzed using the log rank test and Kaplan-Meier analysis. CFU, leukocytes and TNF in the four mouse strains at different time points were compared with repeated-measures analysis of variance (ANOVA).

## Results

### Survival of TLR2^-/-^, CD14^-/- ^and TLR2^-/-^/CD14^-/-^mice after antibiotic therapy

**Figure 1:**
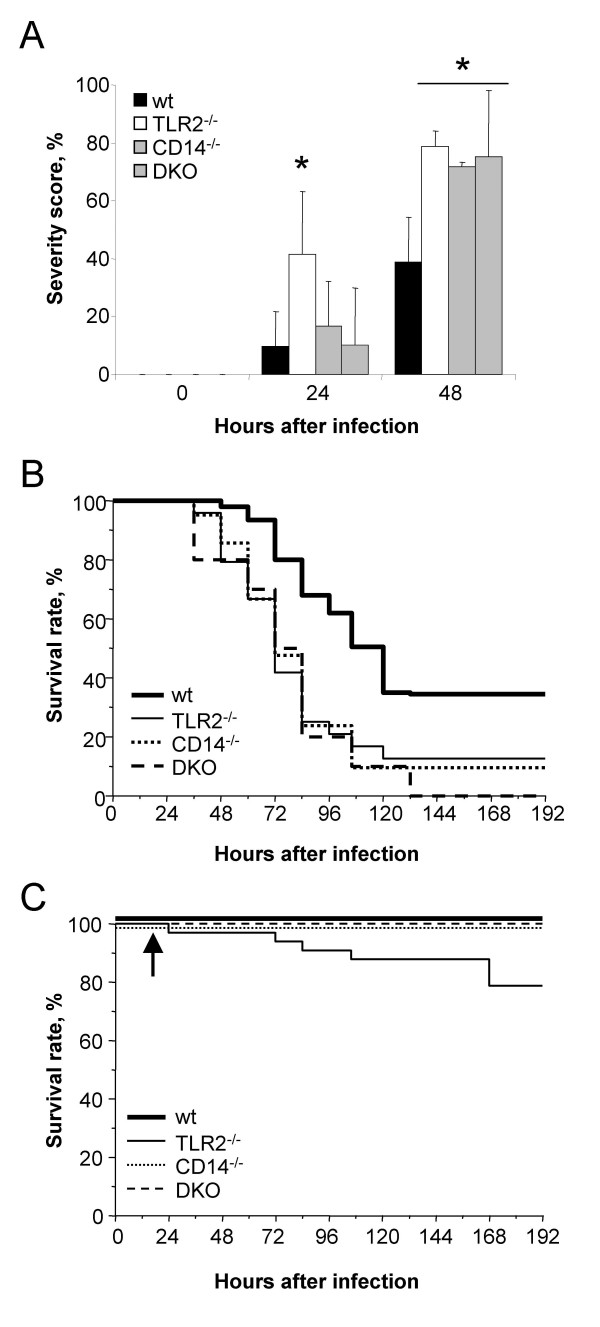
**A) **Percentage of wt (n = 40), TLR2^-/-^(n = 35), CD14^-/- ^(n = 29) and TLR2^-/-^/CD14^-/- ^(n = 31) mice showing high severity score (including score higher than 2.5) after infection with *S. pneumoniae*. Mean ± SD of four independent experiments (*p *< 0.02, ANOVA). **B) **Mortality from untreated pneumococcal meningitis: wt (n = 33), TLR2^-/- ^(n = 24), CD14^-/- ^(n = 21) and TLR2^-/-^/CD14^-/- ^(n = 10) mice. **C) **Effect of antibiotic treatment in wt (n = 15), TLR2^-/- ^(n = 15), CD14^-/- ^(n = 9) and TLR2^-/-^/CD14^-/- ^(n = 9) mice. Arrow indicates start of ceftriaxone therapy.

**Figure 2:**
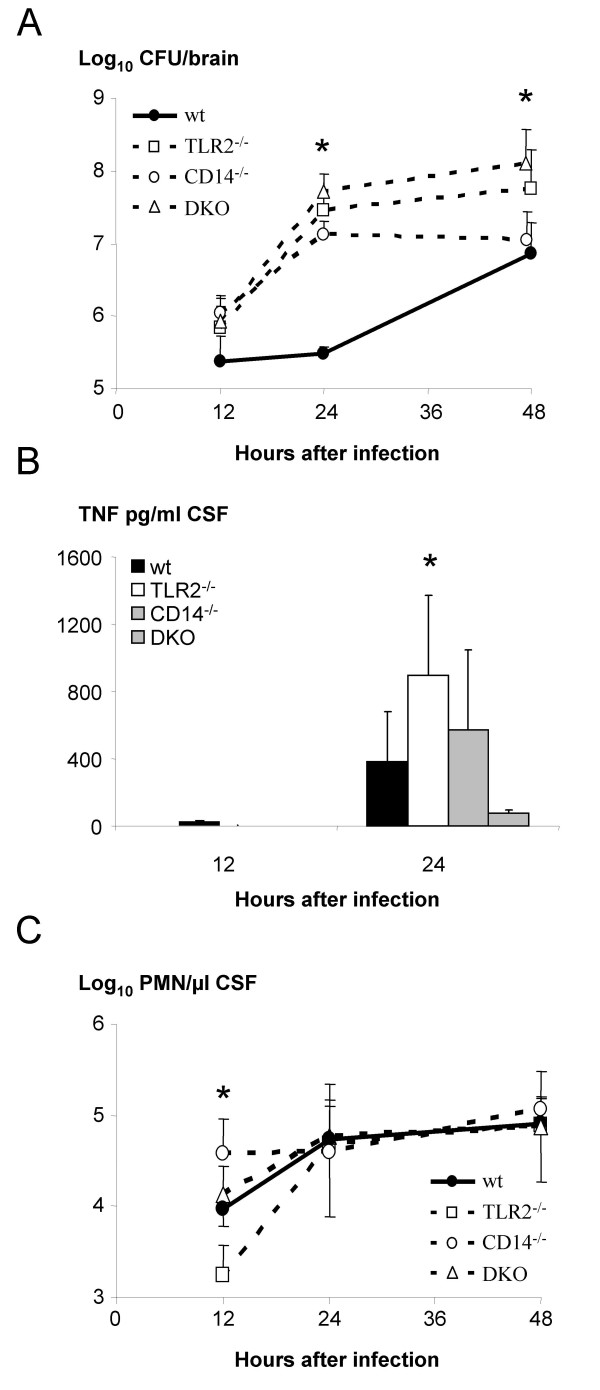
**A) **Bacterial numbers in brain 12, 24 and 48 h after infection with 3 × 10^3 ^CFU of *S. pneumoniae *in wt (n = 18), TLR2^-/- ^(n = 18), CD14^-/- ^(n = 17) and TLR2^-/-^/CD14^-/- ^(n = 6) mice. Median values and 25–75% spread (*p *< 0.02, ANOVA). **B) **TNF levels in CSF in wt (n = 21), TLR2^-/- ^(n = 13), CD14^-/- ^(n = 14) and TLR2^-/-^/CD14^-/- ^(n = 6) mice 12 and 24 h after infection. Mean values ± SD (*p *< 0.02, ANOVA). **C) **Leukocyte numbers in CSF in wt (n = 15), TLR2^-/- ^(n = 10), CD14^-/- ^(n = 14) and TLR2^-/-^/CD14^-/- ^(n = 6) mice 12 to 48 h after infection. Median values (*p *< 0.02, ANOVA).

TLR2^-/-^, CD14^-/-^and C57BL/6 (wt) mice used in this study were described in previous published papers [6,18]. The early course of meningitis differed in the three knockout strains. After 24 h, a large fraction of TLR2^-/- ^mice (41%) showed severe clinical symptoms, whereas in wt, CD14^-/- ^and TLR2^-/-^/CD14^-/- ^mice, only a small proportion of mice (less than 16%) was severely sick (Fig. 1A). After 48 h, all knockout strains showed significantly more animals with high severity scores than wt mice (Fig. 1A). Accordingly, survival was significantly shortened in all knockout compared to wt mice (*p *< 0.02 in log-rank test; Fig. 1B). Antibiotic treatment is efficient in mouse [24] and human meningitis [25], if treatment starts early. Accordingly mice were treated with ceftriaxone for 5 days starting 18 h after infection. While wt, CD14^-/- ^and TLR2^-/-^/CD14^-/- ^mice were rescued (Fig. 1C), antibiotics were insufficient in TLR2^-/- ^mice and 21.2% of TLR2^-/- ^mice died (*p *< 0.05, compared to the other mouse strains). Most likely, antibiotics are less efficient under conditions of established, deleterious inflammation, which follows accumulation of bacteria. In our study the bacterial load and consequently the extent of inflammation may have influenced the antibiotic response. Therefore these parameters were compared in the different mouse strains. Bacterial load and TNF in TLR2^-/-^, CD14^-/- ^and TLR2^-/-^/CD14^-/- ^mice was assessed simultaneously in all knockout mice after infection and values in knockout strains were compared to those in wt mice. Bacterial numbers in CSF were similar in all strains 24 h after infection (data not shown), indicating that planctonic bacteria recovered in CSF did not represent an early pathogenetic factor, which contributed to the more severe clinical picture of the TLR2^-/- ^strain. In contrast CFU numbers collected in brain homogenates were higher in all knockout than in wt mice 24 h after infection (*p *< 0.05, Fig. 2A). After 48 h, values in TLR2^-/-^/CD14^-/- ^mice remained higher than in the other strains (*p *< 0.01, Fig. 2A). TNF levels in CSF were barely detectable after 12 h, but after 24 h TLR2 deficiency was accompanied by a stronger inflammation, recorded as higher TNF level in CSF (Fig. 2B) as compared to CD14^-/- ^and to wt mice. Mice deficient in both TLR2 and CD14 showed hardly any elevation of TNF with values remaining below 100 pg/ml. After 48 h all knockout mice showed low level of TNF (below 100 pg/ml) in CSF. Since in meningitis, TNF in CSF is mainly derived from infiltrating cells, the extent of leukocyte infiltration might have determined TNF levels in the four strains. However, compared to wt mice, TLR2^-/- ^mice presented even a reduced influx (Fig. 2C), CD14^-/- ^mice had an increased influx and TLR2^-/-^/CD14^-/- ^mice had similar leukocyte numbers as wt mice at 12 h after infection. Twenty-four and 48 h after infection, the values were similar in all mouse strains (Fig. 2C). These results indicate, that the control of TNF and leukocyte migration by TLR2 and CD14 were not linked, and high TNF was produced in TLR2^-/- ^mice despite low leukocyte numbers.

Our results show an effect on bacterial clearing of both TLR2 and CD14, a TNF-downregulating effect of TLR2 and an opposite effect of TLR2 and CD14 respectively on early leukocyte migration.

### Effect of TACE inhibitor on meningitis in TLR2^-/- ^and CD14^-/- ^mice

**Figure 3:**
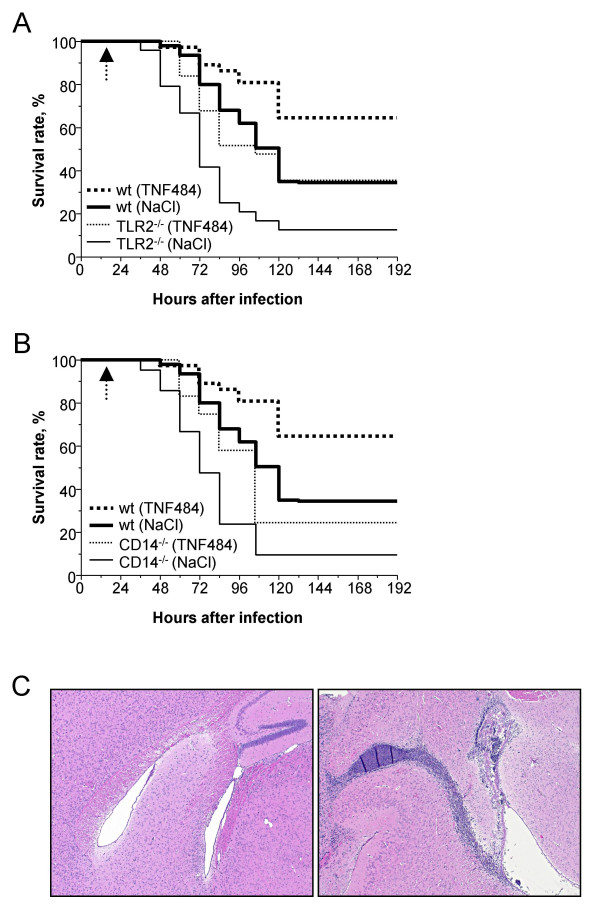
Effect of TACE inhibitor (TNF484) treatment in wt, TLR2^-/-^and CD14^-/-^mice on outcome of pneumococcal meningitis. **A) **Survival rate in wt untreated (n = 25, NaCl) and treated (n = 37, TNF484), *p *< 0.05; TLR2^-/- ^untreated (n = 25, NaCl) and treated (n = 25, TNF484), *p *< 0.05; wt untreated and TLR2^-/- ^treated: *p *> 0.05. **B) **Survival rate in wt untreated (n = 25, NaCl) and treated (n = 37, TNF484), *p *< 0.05; CD14^-/- ^untreated (n = 21, NaCl) and treated (n = 12, TNF484), *p *< 0.05. Dashed arrow indicates start of TNF484 therapy. **C) **HE-stained sections of brains from wt mice 24 h after infection with (left panel) or without (right panel) TNF484 treatment. One representative example is shown for each group.

TLR2^-/-^mice could not be rescued with antibiotics and showed excess TNF in CSF. Therefore, the treatment of choice in TLR2^-/- ^mice was an anti-inflammatory adjunctive drug. Based on our earlier studies with TNF484 in neonatal rat pneumococcal meningitis [26], we confirmed in our mouse meningitis model, that TNF484 treatment starting 12 h after infection reduced CSF infiltrating leukocytes in infected mice by 50% from 6.38 × 10^4 ^± 5.88 × 10^4 ^to 3.22 × 10^4 ^± 1.82 × 10^4 ^leukocytes/μl CSF, respectively. Histology confirmed the reduction of leukocyte infiltration; in TACE-treated brain no leukocytes could be detected (Fig. 3C, left), whereas in untreated infected brains massive leukocyte infiltration was detected (Fig. 3C, right), MMP-9 in brain homogenates of infected and TNF484 – treated mice was reduced to 10% of the value found in infected mice, respectively and TNF in CSF to non-measurable values (data not shown). TNF484 treatment did not rescue mice to 100% survival, but mortality was significantly delayed in both knockout and wt mice (*p *< 0.05). In TLR2^-/-^(Fig. 3A) and CD14^-/- ^(Fig. 3B) animals, this treatment significantly slowed death to the rate observed in untreated wt mice. Survival of wt mice was also significantly prolonged by TNF484 treatment (*p *< 0.05), which indicates a better outcome in mice receiving anti-inflammatory therapy.

### Effect of antibiotics and TNF484 combined in TLR2^-/- ^mice

**Figure 4:**
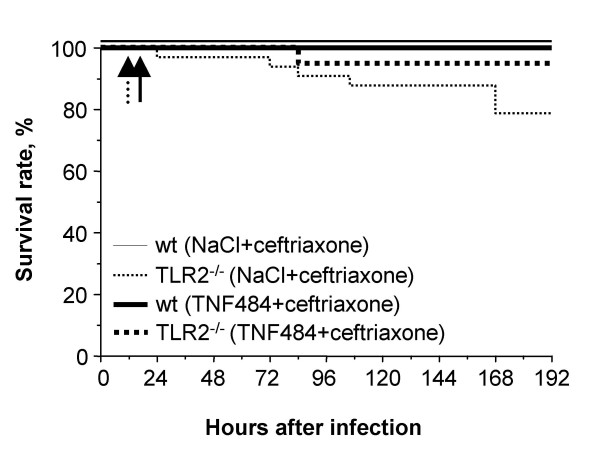
Efficacy of TACE inhibitor (TNF484) and antibiotic (ceftriaxone) treatments on outcome of wt and TLR2^-/- ^*S. pneumoniae *infected mice. Survival rate in wt (n = 15) and TLR2^-/- ^(n = 15) treated with ceftriaxone alone, and in wt (n = 19) and TLR2^-/- ^(n = 33) treated with TNF484 and ceftriaxone combined. Dashed and solid arrows indicate start of twice daily therapy with TNF484 and ceftriaxone, respectively.

Wt, CD14^-/-^and TLR2^-/-^/CD14^-/-^mice, but not TLR2^-/-^mice were rescued by antibiotics. According to the individual effects of ceftriaxone and TNF484 in TLR2^-/-^mice (Fig. 1C and 3A), combining both treatments was expected to result in an additive effect. Therefore, efficacy of the combined TNF484 and antibiotic therapy on outcome of TLR2^-/- ^mice was assessed. Two groups of TLR2^-/-^mice treated either with ceftriaxone alone or with ceftriaxone combined with TNF484 were compared. TLR2^-/-^mice receiving combined treatment all survived, this was a significantly better outcome compared to TLR2^-/-^mice, which received ceftriaxone alone (p < 0.05) (Fig. 4). DKO mice were not treated with TACE inhibitor since they developed a weak meningeal inflammation no effect on TNF could be expected.

## Discussion

This study has compared outcome and treatment effects of antibiotics and TACE inhibitor TNF484 during pneumococcal meningitis in mice lacking the pattern recognition receptors TLR2 and/or CD14. We found that wt mice with untreated *S. pneumoniae *meningitis lived longer than infected mice with a genetic deletion of TLR2 or CD14 or both TLR2 and CD14. The time to death was similar in all untreated KO strains. However, antibiotics rescued all wt, CD14^-/-^and TLR2^-/-^/CD14^-/- ^mice and only 79% of the TLR2^-/- ^mice. This indicates that different mechanisms led to earlier death in the 3 KO mouse strains. TLR2^-/- ^mice presented earlier a more severe disease with higher TNF in CSF than the other strains, thus in these animals death despite antibiotic therapy was most likely due to increased inflammation. Accordingly, the treatment of choice in TLR2^-/- ^mice was an anti-inflammatory adjunctive drug. Based on our earlier studies with TACE inhibitor TNF484 in neonatal rat pneumococcal meningitis [26], we first confirmed that TNF484 used at 1 mg/kg twice daily blocked TNF and MMP-9 in CSF of infected mice. TACE inhibitor treatment was able to reduce inflammation in all mouse strains studied. Wt mice, which had a longer survival if untreated, were similarly improved as TLR2-and CD14-deficient animals, which had a shorter survival if untreated. Since infected TLR2^-/-^mice were not rescued by administration of either antibiotics or TACE inhibitor alone, combined treatment was applied and resulted in 95% survival in this mouse strain. It can be hypothesized that TNF484 treatment reduced the inflammation which was induced by the cell wall components released during ceftriaxone therapy [27]. In an earlier study, penicillin treatment of *S. pneumoniae *led to release of inflammatory products [28]. Since antibiotic-induced *S. pneumoniae *fragments activated host cells via TLR2 [29], it is unlikely that in TLR2^-/- ^mice TNF484 treatment reduced the inflammation, which was induced by the cell wall components released during ceftriaxone therapy [27].

To elucidate the reasons for the differential treatment response in the 4 mouse strains, bacterial load, CSF leukocyte numbers and TNF, which are the major determinants of outcome, were studied. CFU in CSF are derived from planctonic bacteria, they were not different early in disease among the different mouse strains. Since we observed that pneumococci are strongly adherent to plexus epithelia [30], bacterial load in brain homogenates was evaluated. 24 h after infection brain bacterial load was similarly increased in all the KO strains compared to C57BL/6 mice. We have previously investigated the effector mechanism of TLR2 upon bacterial clearance. Early increased brain bacterial load in TLR2^-/- ^mice [6] was associated with a stronger pneumococcal adherence to plexus choroideus epithelial cells in TLR2-deficient brains [30]. In addition, we found phagocytosis of pneumococci delayed and oxidative killing reduced in TLR2^-/- ^compared to wt granulocytes *in vitro *(14). These results are in agreement with impaired phagosome maturation, as described in TLR2-deficient macrophages after ingestion of killed bacteria [31,32]. Thus TLR2 appears to improve bacterial clearance by reducing adherence and accelerating pathogen uptake. In contrast, we found no early direct effect of CD14 upon bacterial numbers *in vivo *[18] and no effect upon pneumococcal phagocytosis of CD14 *in vitro *(unpublished results). Nevertheless after 48 h of infection TLR2^-/-^/CD14^-/-^mice had higher concentrations of bacteria in both CSF and brain than either TLR2^-/-^or CD14^-/-^mice. This indicates that TLR2 has a primary effect upon bacterial clearance, which is supported by CD14.

We previously described a delayed early leukocyte recruitment to CSF in TLR2^-/- ^mice [6] and an accelerated leukocyte immigration in CD14^-/- ^compared to wt mice [18] (Fig. 2C). These findings imply that the early high TNF levels in TLR2^-/- ^mice were not related to high leukocyte numbers in CSF. Instead, we found a NF-κB-dependent excess of pro-over anti-inflammatory cytokine mRNA with an increased TNF and a decreased IL-10 transcription and increased TNF protein in brain leukocytes at the origin of early increased TNF in TLR2^-/- ^mice [15]. In contrast, in CD14^-/-^mice no such changes were observed according to our previous study [18]. Interestingly TLR2/CD14 KO mice produced less TNF than wt mice, which indicates that besides TLR2 as the major regulator, CD14 participates in a temporally and locally coordinated action in controlling TNF during pneumococcal infection.

## Conclusion

Our treatment study compares the host response mediated by TLR2 and CD14 in meningitis, and illustrates the requirement and success of adjuvant therapy, under conditions of harmful inflammation in TLR2-deficient mice.

## Competing interests

The author(s) declare that they have no competing interests.

No organization is financing the manuscript, and there cannot be any financial gain or loss from this publication. The TACE inhibitor (TNF484) used in the present study is not foreseen for preclinical or clinical development, or marketing.

## Authors' contributions

HE performed the experiments together with SLL.

SLL performed statistical analysis of the survival curves.

UN had the contract for the TNF484 with SLL and advised for dosages and therapy with TNF484.

RML lead the project, supervised HE and wrote the manuscript.

All the authors read and approved the final manuscript.

## Pre-publication history

The pre-publication history for this paper can be accessed here:


